# The Fragmented QRS Complex in Lead V_1_: Time for an Update of the Athlete’s ECG?

**DOI:** 10.1007/s12265-023-10448-9

**Published:** 2023-10-31

**Authors:** Marco Vecchiato, Giulia Quinto, Nicola Borasio, Stefano Palermi, Giampaolo Berton, Francesca Battista, Andrea Gasperetti, Andrea Ermolao, Daniel Neunhaeuserer

**Affiliations:** 1 Sports and Exercise Medicine Division, Department of Medicine, University Hospital of Padova, Via Giustiniani 2, 35128 Padua, Italy; 2 Public Health Department, University of Naples Federico II, 80131 Naples, Italy; 3 University of Padova, Department of Medicine, Via Nicolò Giustiniani, 2, 35128 Padua, Italy; 4 Division of Cardiology, Ospedale Alto Vicentino, 36014 Santorso (VI), Italy

**Keywords:** QRS fragmentation, Athlete’s heart, Exercise testing, Right ventricle, Ventricular arrhythmias, Sports

## Abstract

Differentiating between ECG patterns related to athletes' heart remodeling and pathological findings is a challenge in sports cardiology. As the significance of fragmented complex in athletes remains uncertain, this study aimed to assess the presence of fragmented QRS in lead V_1_ (fQRSV_1_) among young athletes and its association with heart adaptations and arrhythmias. Young athletes referred for annual pre-participation screening receiving a maximal exercise testing and transthoracic echocardiography from January 2015 to March 2021 were included. The study included 684 young athletes. The prevalence of fQRSV_1_ was 33%. Subjects with fQRSV_1_ had higher exercise capacity and indexes of right ventricular function and remodeling. Among highly trained athletes, the fQRSV_1_ group demonstrated also increased left ventricular wall thickness. No significant association existed between fQRSV_1_ and exercise-induced arrhythmias, even in highly trained athletes. The high prevalence of fQRSV_1_ in young athletes is associated with training-induced heart adaptations but not exercise-induced ventricular arrhythmias.

**Graphical Abstract:**

The fragmented QRS complex in lead V_1_ in young athletes. PPS = preparticipation screening; EST = exercise stress test; fQRSV_1_ = fragmented QRS in lead V_1_; PSBs = premature supraventricular beats; PVBs = premature ventricular beats.

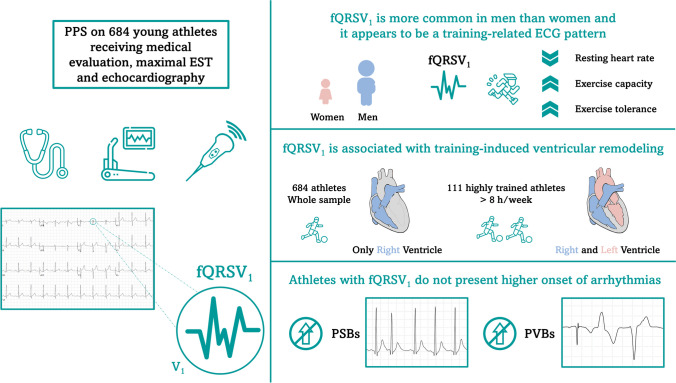

**Supplementary Information:**

The online version contains supplementary material available at 10.1007/s12265-023-10448-9.

## Introduction

Pre-participation screening in young athletes was launched in several countries in order to prevent sports-related cardiac events. Resting 12-lead electrocardiogram (ECG) is one of the most efficient tools to evaluate apparently healthy subjects before starting competitive sports activity [1].

In the last few years, a scientific challenge addressed the identification of ECG patterns typically associated with athletes’ heart remodelling, which may allow to differentiate from potential pathological findings [2]. Indeed, regular exercise training is associated with cardiac chamber size adaptation and increased vagal tone which both could influence resting ECG [3, 4]. Athletes’ heart remodelling was described also in young pre-adolescent athletes with increased right ventricular (RV) and left ventricular (LV) mass and chamber dimensions [5–7].

Recently, some studies explored a new resting ECG pattern in young and adult athletes, i.e. the fragmented QRS complex (fQRS), especially in anterior leads [8, 9]. fQRS was described as a surrogate marker reflecting a minor intra-ventricular conduction delay and it is defined as quadriphasic QRS complex (RSR’S’ pattern) or the presence of notched R or S waves, with a QRS duration < 120 ms [10]. In the general population, fQRS is a common finding and it has not been associated with an increased risk of mortality in subjects without known cardiac disease [11]. In athletes, its prevalence seems to be higher than in non-athletes and the fQRS in lead V_1_ (fQRSV_1_) might be related to training-induced RV remodelling in adult athletes [8]. However, less is known for young athletes and no data is available regarding the association between fQRSV_1_ and exercise-induced ventricular arrhythmias.

Therefore, our study aimed to evaluate the presence of the fQRSV_1_ pattern in the young athletic population and its relationship with training-associated structural heart adaptations and exercise-induced arrhythmias.

## Materials and Methods

### Study Sample and Protocol

This retrospective study consecutively enrolled all young athletes referred for second-line evaluation during the annual pre-participation screening at the Sports and Exercise Medicine Division of the University Hospital of Padova, between January 2015 and March 2021 [12]. Exclusion criteria were age > 18 years, known cardiac disease (congenital heart disease, cardiomyopathies, and arrhythmic syndromes), symptoms and ECG abnormalities. Data regarding medical history, physical examination, ECG characteristics, exercise testing, and transthoracic echocardiography were collected for each participant [13]. The local ethics committee approved this study (Code 129n/AO/21 – date 22.04.2021) and written informed consent was obtained from the parents of all athletes.

### Medical History, Physical Examination and Exercise Testing

Anamnestic data, including family and personal history, were assessed through a reproducible and standardised interview. The sport practiced was evaluated qualitatively through the predominant component of structured exercise, according to the 2020 ESC Guidelines on sports cardiology and exercise in patients with cardiovascular disease and quantitatively based on weekly hours of training [1].

Before physical examination, anthropometric parameters including height and body weight were assessed. In addition, systolic and diastolic blood pressure (SBP and DBP) were measured at rest, at peak exercise as well as during the recovery phase.

The maximal exercise test was conducted on a treadmill with a standardised incremental ramp protocol [14]. Criteria of exhaustion were a Borg rating of perceived exertion (RPE) ≥ 18/20 associated with a maximal heart rate (HR) ≥ 85% of predicted per age. Exercise capacity was evaluated with the metabolic equivalents of task (METs) reached during testing. The presence of arrhythmias was analysed, evaluating morphology and complexity. Arrhythmias were classified as supraventricular and ventricular premature beats and the latter as common and uncommon depending on their characteristics in accordance with the current interpretation recommendations for athletes [15, 16].

### ECG Analysis

Each participant was analysed with a 12-lead ECG trace registered at rest in the supine position and continuously monitored during exercise and recovery. All patients underwent a maximal exercise test. The recovery phase was monitored for at least 4 min.

The instrument used was a GE CASE V6.51 with a sliding speed of 25 mm/s and a calibration of 10 mm/mV.

**Fig. 1:**
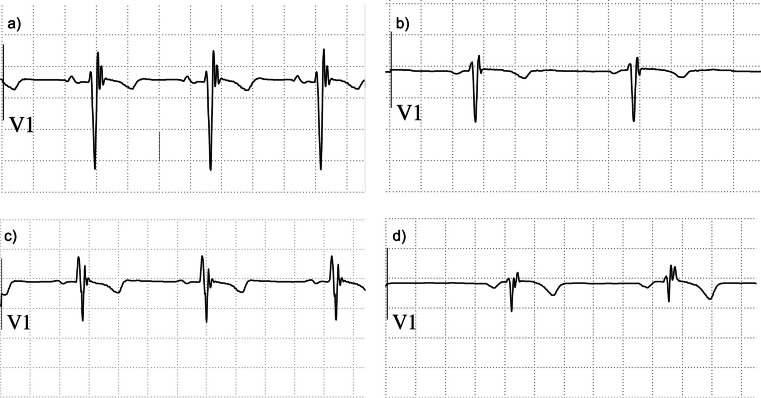
Fragmented QRS patterns in lead V_1_ in young athletes. **a**) 16 years, athletics; **b**) 13 years, dancing; **c**) 13 years, karate; **d**) 8 years, horse riding

The QRS complex pattern in lead V_1_ was evaluated as follows:—normal QRS, defined as an RS pattern with a duration < 110 ms;—incomplete right bundle branch block (iRBBB), defined as a rSr’ pattern with a < 120 ms duration;—complete right bundle branch block (RBBB), defined as a rSr’ pattern with a duration ≥ 120 ms;—fQRSV_1_ defined as a quadriphasic QRS complex (rSr’s’ pattern) with a duration < 120 ms (Fig. 1) [8].

All ECGs were reviewed by two independent physicians (M.V. and G.Q.) and patterns were digitally evaluated by zooming in the interested lead, i.e. V_1_.

### Transthoracic Echocardiography Analysis

M‐mode, two‐dimensional, and Doppler echocardiographic examinations were performed using a multi‐hertz sector, 2‐4 MHz probe‐equipped machine (Vivid 7 Pro, GE Healthcare, Chicago, Illinois, USA). Cardiac structural and functional measurements were obtained according to European guidelines [17]. Left ventricular end-diastolic diameter (LV EDD), left ventricular end-systolic diameter (LV ESD), septal wall thickness (SWT), posterior wall thickness (PWT), aortic bulb diameter, LV mass, left atrial (LA) volume, right ventricular end-diastolic diameter (RV EDD), tricuspid annular plane systolic excursion (TAPSE) and right ventricular outflow tract diameter (RVOT) were determined. LV mass was calculated using the formula described by Devereux et al. [18]. Relative wall thickness (RWT) was determined by the equation RWT = 2 × PWT/LV EDD. LV geometry was defined according to RWT and LV mass [19]. All examinations were performed by the same operator, an ultrasonography‐experienced cardiologist.

### Statistical Analyses

Statistical analyses were performed using Statistical Package for Social Science (SPSS Inc., Chicago, IL, USA; ver. 26). The Kolmogorov–Smirnov test was used to evaluate the normal distribution of all parameters. Continuous variables are expressed as mean ± standard deviation or median (inter-quartile range) and comparisons between subgroups were performed with the Student T test or Wilcoxon-Mann–Whitney test, for normally and non-normally distributed variables, respectively. The relationship between continuous variables was evaluated by Pearson’s or Spearman’s correlation coefficients according to the distribution of the parameters. Categorical variables were expressed as frequencies/percentages and compared between groups using Pearson’s chi-squared test. All reported probability values are two-tailed and a value of *p* < 0.05 was considered statistically significant.

## Results

**Fig. 2:**
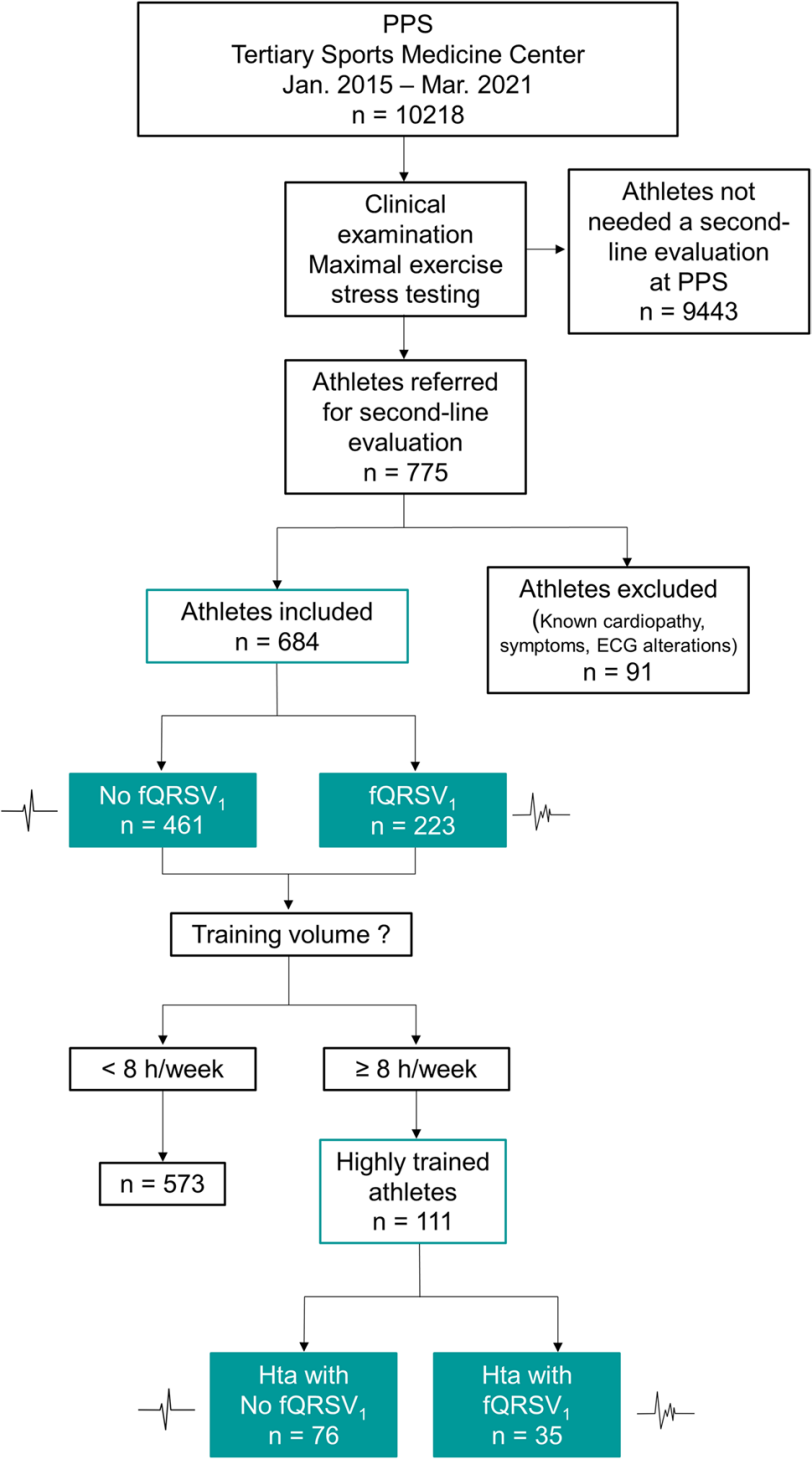
Flow chart of the study. PPS = preparticipation screening; fQRSV_1_ = fragmented QRS in lead V_1_; Hta = highly trained athletes

A total of 684 young athletes (36% females) with a mean age of 14.87 ± 1.96 years were included (Fig. 2). The clinical baseline characteristics of the study sample are shown in Table 1. The overall prevalence of fQRSV_1_ was 33% with more male than female athletes presenting this pattern (78% vs 22%; *p* < 0.001). 83 athletes presented iRBBB (12%) and only 4 RBBB (0.5%). 19% of the total athletes presented fQRS only in the V_1_ lead, 8% in both V_1_ and V_2_ leads. The presence of fQRS pattern in the other leads was investigated and classified as follows: septal (V_1_, V_2_ leads): 34%; inferior (II, III, aVF leads): 7%; lateral (V_5_, V_6_, I, aVL leads): 10%; anterior (V_3_, V_4_ leads): 2%.

**Table 1:** Young athletes with and without fQRSV_1_

	All (*n* = 684)	No fQRSV_1_ (*n* = 461)	fQRSV_1_ (*n* = 223)	*p*
Age (years)	14.87 ± 1.96	14.94 ± 1.97	14.75 ± 1.94	0.204
Gender (female%)	247 (36%)	199 (43)	48 (22)	** < 0.001**
Weight (kg)	59.44 ± 12.91	59.26 ± 12.72	59.83 ± 13.31	0.480
Height (m)	1.67 ± 0.11	1.67 ± 0.11	1.68 ± 0.12	0.790
BMI (kg/m^2^)	21.09 ± 3.03	21.12 ± 2.97	21.01 ± 3.16	0.349
BSA (m^2^)	1.66 ± 0.22	1.65 ± 0.22	1.68 ± 0.23	0.171
Sport category				0.091
Endurance (%)	75 (11)	52 (11)	23 (10)	
Power (%)	104 (15)	77 (17)	27 (12)	
Mixed (%)	477 (70)	309 (67)	168 (76)	
Skilled (%)	28 (4)	23 (5)	5 (2)	
Training (h/week)	6.27 ± 2.07	6.26 ± 2.05	6.29 ± 2.10	0.924

Anthropometric and clinical parameters of the study population. Data are expressed as mean ± standard deviation or frequencies (percentage)

*fQRSV*_*1*_ fragmented QRS in lead V_1_, *BMI* body mass index, *BSA* body surface area

Bold entries is for *p *< 0.05

Athletes participated in 44 different sports disciplines and data revealed that subjects with fQRSV_1_ did not differ by sports category (endurance vs power vs mixed vs skill) or training volume per week. The full list of sports practised has been included in Supplementary Table 1.

Resting and exercise test ECG parameters as well as echocardiographic data are reported in Table 2. Subjects with fQRSV_1_ showed a significantly wider QRS interval (*p* = 0.004), lower QTc values (*p* < 0.001) and inferior HR at rest (*p* = 0.001) while resting and peak blood pressure values showed no difference between groups. Peak exercise capacity expressed in METs and exercise duration were higher in subjects with fQRSV_1_ (*p* = 0.002 and *p* = 0.023, respectively). Furthermore, athletes with fQRSV_1_ showed a higher indexed RV EDD (*p* = 0.019) and TAPSE (*p* = 0.013). No difference in the remaining echocardiographic parameters was detected. Subjects with fQRSV_1_ did not show an increased occurrence of supraventricular or ventricular arrhythmias, regardless of morphology and complexity (neither for isolated nor for repetitive events). During the mean follow‐up time of 4.57 ± 2.71 years, no major cardiovascular events were recorded.

**Table 2:** Electrocardiographic, exercise test and echocardiographic parameters

	No fQRSV_1_ (*n* = 461)	fQRSV_1_ (*n* = 223)	*p*
ECG			
PQ (ms)	138.28 ± 22.04	137.84 ± 23.10	0.904
QRS interval (ms)	75.32 ± 32.32	76.24 ± 10.58	**0.004**
QTc (ms)	395.44 ± 25.87	388.64 ± 25.48	** < 0.001**
QRS voltage (mm)	31.65 ± 9.12	30.27 ± 9.38	0.054
Resting Parameters			
HR rest (bpm)	70.08 ± 12.45	66.98 ± 12.27	**0.001**
SBP rest (mmHg)	112.34 ± 11.85	111.46 ± 12.48	0.213
DBP rest (mmHg)	63.69 ± 8.67	63.33 ± 8.56	0.458
Exercise Parameters			
HR peak (bpm)	189.63 ± 9.28	188.33 ± 8.18	0.081
SBP peak (mmHg)	161.56 ± 22.14	164.30 ± 25.14	0.099
DBP peak (mmHg)	47.29 ± 11.82	47.93 ± 12.10	0.556
METs peak	17.31 ± 2.77	17.95 ± 2.74	**0.002**
Exercise time (s)	434.50 ± 55.53	441.43 ± 50.08	**0.023**
Arrhythmias			
PSBs (*n*, %)	136 (36)	69 (31)	0.722
PVBs isolated (*n*, %)	203 (44)	95 (43)	0.743
Common morphology* (*n*, %)	157 (34)	69 (31)	0.436
Uncommon morphology** (*n*, %)	52 (11)	29 (13)	0.529
PVBs repetitive‡ (*n*, %)	21 (5)	11 (5)	0.848
Echocardiography			
Indexed LV EDD (mm/m^2^)	28.13 ± 3.52	28.15 ± 3.21	0.759
Indexed LV ESD (mm/m^2^)	17.07 ± 2.49	17.28 ± 2.45	0.322
LVEF (%)	69.20 ± 6.19	68.56 ± 6.58	0.203
SWT (mm)	8.25 ± 1.31	8.37 ± 1.34	0.125
PWT (mm)	7.33 ± 1.27	7.49 ± 1.31	0.090
Aortic bulb (mm)	24.47 ± 2.99	24.81 ± 3.09	0.361
Indexed LA volume (ml/m^2^)	29.11 ± 3.67	29.10 ± 3.75	0.943
Indexed LV mass (mg/m^2^)	71.70 ± 18.20	72.41 ± 15.65	0.185
Indexed RV EDD (mm/m^2^)	19.81 ± 3.20	20.42 ± 3.12	**0.019**
TAPSE (mm)	23.75 ± 2.97	24.33 ± 3.09	**0.013**
Indexed RVOT (mm/m^2^)	16.23 ± 2.48	16.33 ± 2.23	0.344

ECG, exercise test, and echocardiography parameters of the study population (*n* = 684) grouped by the presence or absence of a fQRSV_1_ ECG pattern. Data are expressed as mean ± standard deviation or frequencies (percentage)

*fQRSV*_*1*_ fragmented QRS in lead V_1_, *QRS voltage* voltage criteria for left ventricular hypertrophy (Sokolow-Lyon index), *HR* heart rate, *SBP* systolic blood pressure, *DBP* diastolic blood pressure, *PSBs* premature supraventricular beats, *PVBs* premature ventricular beats, *LV* left ventricle, *LA* left atrium, *EDD* end diastolic diameter, *ESD* end systolic diameter, *LVEF* left ventricular ejection fraction, *SWT* septal wall thickness, *PWT* posterior wall thickness, *RV* right ventricle, *TAPSE* tricuspid annular plane systolic excursion, *RVOT* right ventricular outflow tract diameter

^*^Common morphology in athletes: infundibular, fascicular

^**^Uncommon morphology in athletes: atypical right bundle branch block and QRS ≥ 130 ms, left bundle branch block with superior or intermediate axis

^‡^Couplets, triplets, or non-sustained ventricular tachycardia

111 subjects who engaged in at least 8 h of structured exercise training per week were considered “highly trained” athletes and further analyses of this subgroup were performed (Table 3). The overall prevalence of fQRSV_1_ in “highly trained” athletes was 32% with still more men than women presenting this ECG pattern (*p* = 0.029). Moreover, in addition to the differences in RV EDD and TAPSE already highlighted for the whole sample, “highly trained” athletes showed higher SWT and PWT when compared to those with no fQRSV_1_. Furthermore, no difference in arrhythmic events has been revealed between “highly trained” athletes with and without fQRSV_1_.

**Table 3:** fQRSV_1_ in “highly trained” athletes

Athletes ≥ 8 h/week (*n* = 111)	No fQRSV_1_ (*n* = 76)	fQRSV_1_ (*n* = 35)	*p*
Age (years)	14.98 ± 1.91	15.39 ± 2.05	0.243
Gender (female %)	43 (57)	12 (34)	**0.029**
Sport category			0.135
Endurance (%)	24 (32)	13 (37)	
Power (%)	17 (22)	10 (29)	
Mixed (%)	27 (36)	12 (34)	
Skilled (%)	8 (10)	0 (0)	
Training (h/week)	9.62 ± 1.99	9.56 ± 2.98	0.549
METs peak	17.29 ± 2.89	18.04 ± 2.88	0.164
Arrhythmias			
PSBs (*n*, %)	27 (35%)	10 (29%)	0.470
PVBs isolated (*n*, %)	33 (43%)	20 (57%)	0.179
Common morphology* (*n*, %)	24 (32%)	17 (49%)	0.085
Uncommon morphology** (*n*, %)	10 (13%)	4 (11%)	0.799
PVBs repetitive‡ (n, %)	2 (3%)	0 (0%)	0.330
Echocardiography			
Indexed LV EDD (mm/m^2^)	28.10 ± 2.99	27.28 ± 3.59	0.239
Indexed LV ESD (mm/m^2^)	17.31 ± 2.23	16.57 ± 2.69	0.108
LVEF (%)	68.14 ± 6.07	69.06 ± 6.83	0.622
SWT (mm)	7.93 ± 1.06	8.51 ± 1.25	**0.011**
PWT (mm)	7.08 ± 1.13	8.00 ± 1.28	**0.001**
Indexed LV mass (mg/m^2^)	69.09 ± 18.83	71.58 ± 17.20	0.398
Indexed RV EDD (mm/m^2^)	19.98 ± 3.57	21.39 ± 3.49	**0.017**
TAPSE (mm)	23.51 ± 2.50	25.89 ± 3.53	**0.001**
Indexed RVOT (mm/m^2^)	16.64 ± 3.38	17.17 ± 2.92	0.318

“Highly trained” athletes (performing ≥ 8 h/week for the last 6 months) in the study population (*n* = 111) grouped by the presence or absence of a fQRSV_1_ ECG pattern. Data are expressed as mean ± standard deviation or frequencies (percentage)

*fQRSV*_*1*_ fragmented QRS in lead V_1_, *PSBs* premature supraventricular beats, *PVBs* premature ventricular beats, *LV* left ventricle, *EDD* end diastolic diameter, *ESD* end systolic diameter, *LVEF* left ventricular ejection fraction, *SWT* septal wall thickness, *PWT* posterior wall thickness, *RV* right ventricle, *TAPSE* tricuspid annular plane systolic excursion, *RVOT* right ventricular outflow tract diameter

^*^Common morphology in athletes: infundibular, fascicular

^**^Uncommon morphology in athletes: atypical right bundle branch block and QRS ≥ 130 ms, left bundle branch block with superior or intermediate axis

^‡^Couplets, triplets, or non-sustained ventricular tachycardia

In multivariate analysis, fQRSV_1_ was independently associated with the age, gender, and TAPSE in the whole study population (Table 4A). When the multivariate analysis was restricted to “highly trained” athletes, not only TAPSE but also indexed RV EDD and PWT remained independently associated with fQRSV_1_ (Table 4B).

**Table 4:** Multivariate analysis for the prediction of a fQRSV_1_ pattern in the whole study population (A; *n* = 684) as well as in “highly trained” athletes (B; *n* = 111)

A	aOR	95% CI	*p*
Female gender (vs male)	0.310	0.204 – 0.470	** < 0.001**
Age (years)	0.887	0.799 – 0.984	**0.024**
Indexed LV EDD (mm/m^2^)	0.972	0.913 – 1.035	0.377
PWT (mm)	1.041	0.888 – 1.221	0.620
Indexed RV EDD (mm/m^2^)	1.030	0.979 – 1.084	0.248
TAPSE (mm)	1.090	1.028 – 1.155	**0.004**
B	aOR	95% CI	*p*
Female gender (vs male)	0.442	0.131 – 1.494	0.189
Age (years)	1.003	0.747 – 1.346	0.985
Indexed LV EDD (mm/m^2^)	0.958	0.795 – 1.155	0.655
PWT (mm)	1.916	1.150 – 3.192	**0.013**
Indexed RV EDD (mm/m^2^)	1.249	1.051 – 1.484	**0.011**
TAPSE (mm)	1.204	1.015 – 1.428	**0.033**

*fQRSV*_*1*_ fragmented QRS in lead V_1_, *aOR* adjusted odds ratio, *95% CI* 95% confidence interval, *LV* left ventricle, *EDD* end diastolic diameter, *PWT* posterior wall thickness, *RV* right ventricle, *TAPSE* tricuspid annular plane systolic excursion

## Discussion

The aim of the present study was to indagate whether the presence of the fQRSV_1_ ECG pattern might be associated with physiological or pathological heart adaptations, focusing specifically on young athletes.

The main results of this study can be summarised as follows:

1. fQRSV_1_ is a frequent and training-related ECG pattern also in apparently healthy young athletes.
2. fQRSV_1_ pattern is associated with training-induced right and left ventricular remodelling.
3. Subjects with fQRSV_1_ do not present higher onset of arrhythmias at maximal exercise testing compared with subjects with no fQRSV_1_.

Different fQRS morphologies have been described in the past with a heterogeneous prevalence in subjects with and without cardiac disease, strictly depending on the definition and localisation used for the fQRS determination [11, 20, 21]. Moreover, a recent study analysing the prevalence of fQRSV_1_ in adult athletes showed an association with training-induced RV remodelling, proposing to investigate this phenomenon also in younger athletes and evaluate the related arrhythmic risk [8]. For this reason, only fQRSV_1_ having an rSr’s’ with a quadriphasic or higher-phasic pattern in lead V_1_ was considered for this study, in order to eliminate possible confounding with the typical conduction delay in the iRBBB and RBBB. The QRS complex in lead V_1_ was recently detailed for young athletes, distinguishing iRBBB from the *crista supraventricularis* pattern (defined as an rSr´ pattern in lead V_1_ together with a QRS ≤ 100 ms and S wave < 40 ms in I or V_6_), with the latter often misdiagnosed for an iRBBB [22]. The prevalence of the *crista* pattern in young athletes appears to exceed that of iRBBB and does not appear to be associated with RV dilation [22]. Moreover, a recent algorithm for ECG interpretation in children practicing sport was proposed [23], reducing the time cut-off to classify the iRBBB to less than 100 ms, not mentioning the presence of fQRS as borderline or abnormal finding. In addition, iRBBB was classified as borderline ECG finding in children, thus requiring further investigation when associated with right or left axis deviation. The classification for iRBBB used in our study and the consequent need for further investigations followed that adopted in the adult athlete population.

fQRS has been traditionally described as a marker of conduction delay that may represent an abnormal area of the myocardium, thus predisposing to a greater arrhythmic risk in patients with heart disease [21]. Indeed, the underlying substrate of the fQRS could be linked to the presence of a myocardial scar, reflecting the inhomogeneous activation of the ventricles [24, 25]. Some authors described the fQRS as a sign of myocardial perfusion deficit [26], linking this pattern to specific heart diseases like sarcoidosis [27]. Furthermore, it has been shown that a baseline fQRS was associated with an up to three-fold increased risk for major arrhythmic events in patients with Brugada syndrome [28].

However, in recent years, fQRSV_1_ has been described as a frequent ECG pattern in young and healthy athletes, frequently associated with training-induced ventricular remodelling [8, 9]. Given the high demand related to the athletes’ cardiovascular workload, many structural and electrical adaptations can occur, thus explaining common features of the athlete’s heart considered uncommon in the general population [29–31]. Our study’s results align with this physiological interpretation, showing how fQRSV_1_ could be a marker of sport-related ventricular adaptation; indeed, subjects with fQRSV_1_ had higher index RV EDD and TAPSE values, while this pattern was independently associated with the global RV function. Despite no difference in weekly training hours has been detected, athletes with the fQRSV_1_ demonstrated higher exercise capacity and tolerance, indices of improved training adaptations, suggesting that fQRSV_1_ is probably more related to the training intensity rather than the training volume. On the other hand, sports classification did not seem to be a significant modifier in this sporty population of children and adolescents, as also previously demonstrated by Orlandi et al. [9].

Moreover, a subgroup analysis was performed, identifying 111 young athletes with at least 8 h of training per week, defined as “highly trained” athletes, using the most recent definition of athletes and considering current studies on this topic [8, 32]. In this “highly trained” subgroup, athletes with fQRSV_1_ presented markers of RV and LV remodelling, and the pattern resulted independently associated with RV and LV function and adaptation. Current evidence explaining adaptations to high training load in the paediatric heart is few and sometimes with conflicting outcomes [33]. Indeed, if D’Ascenzi et al. described a paediatric physiological training-induced remodelling, especially in RV parameters [5], Rodriguez-Lopez et al. affirmed that cardiac remodelling in young athletes is mainly focused on the left chambers [6]. Thus, considering only studies analyzing fQRS in athletes, Orlandi et al. recently showed how the fQRS pattern was independently associated with LV cardiac mass indices, while a study by Ollitreaut et al. demonstrated that fQRSV_1_ was associated with training-induced RV remodeling [8].

One possible explanation for these conflicting results may be the connection between age and training load. Indeed, it is well known that training volume and intensity increase progressively with the development of children and adolescents [34]. The fQRSV_1_ pattern is associated with structural and functional changes typical of the athlete's heart, specifically evident in the right sections. As training volume increases, cardiac adaptations may amplify, and this is also reflected in surface ECG patterns associated with cardiac remodeling. It is possible that with larger training volumes and loads, the fQRSV_1_ pattern, although always strictly linked to the electrical activity of right sections, is more affected by ventricular adaptations of the left chambers that proportionally exceed the growth of the right chambers.

The presence of a fQRS pattern could complicate the decision-making process regarding eligibility for participation in competitive sports as no sufficient data are yet available providing evidence on the clinical relevance of this ECG characteristic in athletes. Indeed, the relationship between fQRSV_1_ and arrhythmias has never been investigated in healthy young athletes. It is known that fQRS may indicate potentially higher risk in patients with well-established structural heart disease or channelopathy but the relationship between fQRS and major arrhythmias is not fully understood [35]. In our study subjects with fQRSV_1_ presented a similar arrhythmic burden during maximal exercise testing compared to the rest of the subjects, both in terms of the origin (supraventricular or ventricular), morphology, or complexity (isolated or repetitive). fQRSV_1_ does not appear to be a pattern that might predispose to greater arrhythmic risk, supporting its para-physiological relevance. The electrical delay that might be hypothesised from the ECG pattern does not seem to enhance the common mechanisms of the onset of ventricular arrhythmias, not even for those originating from the RVOT [8].

Therefore, fQRSV_1_ appears to be a benign marker of cardiac remodelling, more common in highly trained subjects, and the associated morpho-functional adaptations do not affect the arrhythmic burden, particularly regarding ventricular arrhythmias. For this reason and for the absence of reported major adverse events during the follow-up period, fQRSV_1_ should currently be considered a common unremarkable sign in the athlete’s ECG [36].

### Limitation and Perspectives

To the best of our knowledge, this is the largest study on the fQRS ECG pattern in a population of young athletes and the first to specifically address some open questions in sports cardiology regarding the associated risk of exercise-induced arrhythmias. Nevertheless, there are some limitations to be reported. Our study included only young athletes who underwent echocardiography as second-line investigation during the pre-participation screening for sports eligibility. The indications to perform echocardiography were related to minor diagnostic findings detected during the first-line examination as murmur or ≥ two premature ventricular beats. Nevertheless, to minimize this possible selection bias, athletes with previously known cardiopathies, ECG abnormalities or symptoms were excluded from the study. Moreover, after the second-line investigations, including 24 h Holter ECG (with an exercise session, none of the included athletes was found to be not eligible for competitive sports. Furthermore, our study focused only on standard systolic parameters, while other evaluations such as global longitudinal strain, Doppler tissue imaging, or speckle tracking should be added in future investigations. Newer techniques of RV segmentation could help in the evaluation of the relationship between the fQRSV_1_ pattern and RV remodeling, such as RV strain [37].

## Conclusions

The fQRSV_1_ pattern is a common finding on the resting ECG of apparently healthy young athletes and could be related to training-induced ventricular remodeling. Given the absence of a relationship with training-induced ventricular arrhythmias, these data support the fact that fQRSV_1_ pattern should most likely be considered a benign sign in young athletes.

### Supplementary Information

Below is the link to the electronic supplementary material.

Supplementary file1 (DOCX 18 KB)

## Data Availability

The data that support the findings of this study are available from the corresponding author upon reasonable request.
