# Supplementary material for: The Fragmented QRS Complex in Lead V1: Time for an Update of the Athlete’s ECG?
Source: J Cardiovasc Transl Res. 2023 Oct 31;17(1):24–32. doi: 10.1007/s12265-023-10448-9 (PMC10896913; doi:10.1007/s12265-023-10448-9)
Supplement: Supplementary file 1 — Supplementary file1 (DOCX 18 KB) [file 12265_2023_10448_MOESM1_ESM.docx]

**Supplementary Table 1**

| **Sports discipline** | **Number of athletes** | **Percentage (%)** |
| --- | --- | --- |
| Alpine skiing | 1 | 0.1 |
| Artistic gymnastics | 57 | 8.3 |
| American football | 1 | 0.1 |
| Baseball | 7 | 1.0 |
| Basketball | 134 | 19.6 |
| Boxing | 1 | 0.1 |
| BMX | 1 | 0.1 |
| Classical ballet | 5 | 0.7 |
| Climbing | 1 | 0.1 |
| Cycling | 5 | 0.7 |
| Dancesport | 9 | 1.3 |
| Fencing | 4 | 0.6 |
| Figure skating | 20 | 2.9 |
| Finswimming | 2 | 0.3 |
| Football/Soccer | 167 | 24.4 |
| Golf | 1 | 0.1 |
| Grass hockey | 2 | 0.3 |
| Handball | 2 | 0.3 |
| Heavy lifting | 2 | 0.3 |
| Hockey | 1 | 0.1 |
| Horse riding | 2 | 0.3 |
| Ice skating | 1 | 0.1 |
| In-line skating | 1 | 0.1 |
| Judo | 1 | 0.1 |
| Karate | 7 | 1.0 |
| Kayak | 1 | 0.1 |
| Kick boxing | 4 | 0.6 |
| Mixed martial arts | 1 | 0.1 |
| Motocross | 1 | 0.1 |
| Mountain bike | 1 | 0.1 |
| Rowing | 8 | 1.2 |
| Rugby | 32 | 4.7 |
| Speed skating | 4 | 0.6 |
| Synchronized swimming | 8 | 1.2 |
| Swimming | 29 | 4.2 |
| Table tennis | 1 | 0.1 |
| Taekwondo | 7 | 1.0 |
| Tennis | 12 | 1.8 |
| Track and field | 24 | 3.5 |
| Ultimate frisbee | 4 | 0.6 |
| Volleyball | 109 | 15.9 |
| Water polo | 11 | 1.6 |

The total of the percentages exceeds 100% because some athletes play several competitive sports at the same time. The number of disciplines listed is 42 and not 44 as reported in the manuscript because track and field was considered to have an endurance, power or mixed component depending on the subspeciality practiced.
